# Organic Dairy Cattle: Do European Union Regulations Promote Animal Welfare?

**DOI:** 10.3390/ani10101786

**Published:** 2020-10-01

**Authors:** Eugénie Duval, Marina A.G. von Keyserlingk, Benjamin Lecorps

**Affiliations:** 1 Centre de Recherche sur les Droits Fondamentaux et les Évolutions du Droit (CRDFED, EA 2132), UFR de Droit, Administration Économique et Sociale et Administration Publique, Université de Caen Normandie, Esplanade de la Paix, CS14032, CEDEX 5, 14032 Caen, France; duval.eugenie@gmail.com; 2 Animal Welfare Program, Faculty of Land and Food Systems, University of British Columbia, 2357 Main Mall, Vancouver, BC V6T 1Z6, Canada; nina@mail.ubc.ca

**Keywords:** animal well-being, dairy cows, legislation, law, natural living

## Abstract

**Simple Summary:**

This paper aims to identify improvements and gaps in the specific EU regulations for organic farming and whether they promote higher welfare standards for dairy cattle compared to the “minimum standards” set up for conventional farming. Based on the available scientific evidence, we identified areas in the organic regulations where the welfare status of the animals is improved, but some limitations and gaps exist.

**Abstract:**

Animal welfare is an emerging concept in EU law; with the advent of specific regulations intending to protect animals. The approach taken by European lawmakers is to provide “minimum standards” for conventional farming; argued by some as failing to adequately protect animals. In contrast, the EU organic farming regulations aim to “establish a sustainable management system for agriculture” and promote “high animal welfare standards”. The first aim of this review was to identify key areas where there are clear improvements in quality of life for dairy cattle housed under the EU organic regulations when compared to the conventional EU regulations. Using the available scientific evidence, our second aim was to identify areas where the organic regulations fail to provide clear guidance in their pursuit to promote high standards of dairy cattle welfare. The greater emphasis placed on natural living conditions, the ban of some (but unfortunately not all) physical mutilations combined with clearer recommendations regarding housing conditions potentially position the organic dairy industry to achieve high standards of welfare. However, improvements in some sections are needed given that the regulations are often conveyed using vague language, provide exceptions or remain silent on some aspects. This review provides a critical reflection of some of these key areas related to on-farm aspects. To a lesser extent, post farm gate aspects are also discussed

## 1. Introduction

Providing good welfare should (1) “ensure good physical health and functioning of animals” (2); “minimize unpleasant ‘affective states’ (pain, fear, etc.)” and “allow animals normal pleasures” and, (3) “allow animals to develop and live in ways that are naturals for the species” [1]. However, many common routine management practices in dairy farming fail to consider all three concepts; thus, compromising animal welfare. For instance, many dairy farms report high rates of disease undermining biological functioning (e.g., mastitis: [2]); routinely engage in painful procedures without pain mitigation triggering negative affective states (e.g., disbudding: [3]); and fail to provide opportunities to express highly motivated behaviors (e.g., favoring zero grazing systems: [4]; for cows’ motivation to access pasture, see [5]).

Animal welfare science directed towards farm animals has focused primarily on providing science-based evidence to mitigate the negative effects of housing (i.e., restrictive housing designs) and management practices (i.e., painful procedures) on the animals [6]. Despite these efforts, much of the scientific knowledge identifying best practices has not yet been implemented on farm or integrated into current regulations [7]. Hence, current living conditions on many farms often fail to achieve high welfare standards [8], challenging the idea that farm animals, including dairy cattle, live “a life worth living” [9].

**Figure 1:**
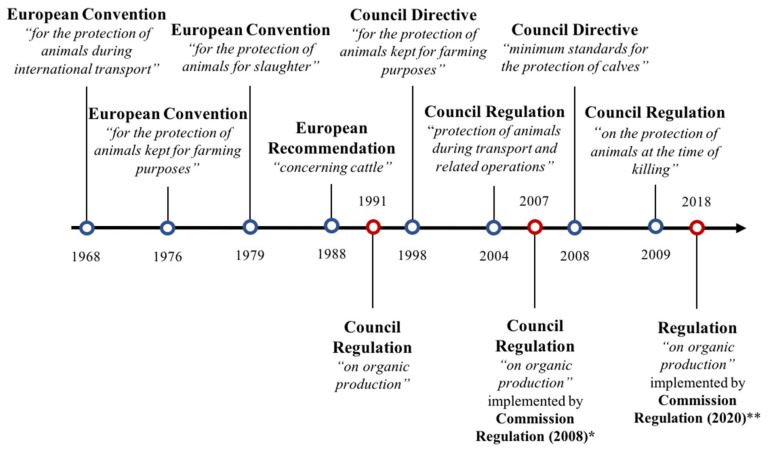
Timeline of European legislation (for conventional and organic farming) with regard to dairy cattle welfare (current as of August 2020). Texts presented above (blue dots) apply to both conventional and organic farming but texts presented below (red dots) apply specifically to organic farms. Council directives have to be transposed by Member States in their national legal systems. In contrast, *regulations* for organic farming and for transportation and slaughtering are more detailed and binding as they are directly applicable in all Member States (no need for national transposition). European Conventions and Recommendations are Council of Europe (CoE) texts—not EU; however, they are a binding part of EU law. * Repeal the texts adopted in 1991 and in force until 1 January 2021. ** Repeal the texts adopted in 2007/2008 and will come into force after 1 January 2021.

The general concept of animal welfare provided by the Treaty on the functioning of the EU [10] considers animals as “sentient beings”, justifying why “the Union and the Member States shall […] pay full regard to the welfare requirements of animals”. The EU law-makers have adopted regulations “laying down minimum standards” for the protection of farm animals. These so-called EU minimum standards are not ambitious regarding the welfare of farm animals [11]. In theory, farmers should “take all reasonable steps to ensure the welfare of animals under their care“ (article 3, Directive 1998 [12]; see [11,13]). However, law-makers have adopted specific rules on a few aspects and stayed silent on most other issues. Therefore, in the case of dairy cattle, these texts allow (or do not explicitly forbid) calves to be individually housed for the first 8 weeks of their life, disbudded without pain mitigation (under 4 weeks old) and zero grazing practices. These practices have raised concerns in the public [14] and according to the available science, challenge the welfare of the animals [15]. In contrast, the EU has adopted more detailed texts specifically aiming at providing higher standards of care for animals in the organic farming sector. Since 1968, many texts have thus been endorsed for both conventional and organic farming (see Figure 1).

Sales of organic goods, including food animal products, have doubled in the EU between 2012 and 2016 [16]. This increase has resulted in a corresponding increase in animals designated as organic; for instance, the number of dairy cows in this sector rose from ~100,000 in 2000 to 860,000 in 2015 (now representing ~4% of the European herd; [17]). Moreover, providing an improved quality of life for farm animals is of increasing concern to consumers of organic products [18,19,20]. In a recent survey, more than 75% of the 28,031 respondents considered that organic food products support higher animal welfare standards [21].

Unlike previous EU regulations related to conventional farming, the organic regulations ([22]; Figure 1) provide detailed rules specifically targeted at promoting “high animal welfare standards and in particular meets animals’ species-specific behavioural needs”. In organic farms, “Husbandry practices, including stocking densities and housing conditions, shall ensure that the developmental, physiological and ethological needs of the animals are met” (art. 1.7.2, 2018/848).

However, within the EU, regulations come through consensus-based deliberations that inherently include some degree of compromise by different stakeholders involved in the decision-making process. These compromises can lead to the adoption of regulations that fall short in their intention to protect the animals, due in part to omissions of fact or the inclusion of vague descriptions [23]. In addition, regulations that are not enforced, or only enforced sporadically, are at great risk of failing to protect animals [11]. The majority of conventional farms are rarely inspected [24], despite some Member States choosing to inspect more frequently than others (e.g., 10% of Swedish farms are inspected every year; [25]). In the case of organic farms, the EU law-makers require all farms to be inspected when seeking organic certification (art. 34, 2018/848) and they are also “subject to a verification of compliance at least once a year” including “a physical on-the-spot inspection”.

The aim of this paper is to critically review the EU organic regulations for dairy cattle and explore whether they result in a meaningful step forward to improve the welfare of dairy cattle. For this, we examined six key areas where there is a body of science available to help guide best practice. For an exhaustive list of the EU conventional and organic regulations concerning dairy cattle production, the reader is referred to the electronic Supplementary Materials (see Tables S1–S22).

Using the minimum standards set for conventional farming as a basis for comparisons, we reviewed the current scientific knowledge in each particular area to identify improvements, limitations and gaps in the organic regulations. We also explored whether some aspects of the organic regulations, that target other objectives of organic farming such as the reduction in use of pharmaceutic agents, may create tensions with respect to animal welfare.

## 2. Physical Mutilations

Most painful management procedures involving physical mutilations of farm animals are addressed within the EU organic regulations. In dairy cattle, physical mutilations include disbudding (i.e., preventing the growth of horns), dehorning (i.e., removing the horns) and tail-docking (i.e., cutting the tail between the sixth and seventh vertebrae). Castration will not be considered as the practice is rarely performed on dairy farms.

Unfortunately, the regulations for conventional farming are not clear regarding physical mutilations. The Council of Europe Recommendation (CoE) [26] forbids tail-docking (except for medical purposes) but allows dehorning and disbudding (anesthesia is mandatory when disbudding and dehorning calves over 4 weeks of age). However, the directives adopted in 1998 [12] and 2008 [27] failed to address physical mutilations, making it unclear whether these rules still apply. There is some evidence indicating that the CoE recommendations are largely ignored; a recent survey in the EU reported that about 60% of calves aged over 2 months old were not given any pain mitigation drugs before or after dehorning [3].

The regulations governing organic farming ban these procedures or state that they may only be performed “on a case-by-case basis and only when those practices improve the health, welfare or hygiene of the livestock or where workers’ safety would otherwise be compromised”. Tail-docking is not mentioned in the listed exceptions in the 2018 organic regulations and thus appears to be banned by default, a position similar to many other countries (see [8]). Thus, only dehorning and disbudding may be performed but under circumstances that guarantee minimal suffering for the animals.

The practices of disbudding and dehorning (please note that the majority of research has focused on disbudding) intend to reduce the risks of injuries to animals and stockpersons caring for the animals (see review by [28]). Disbudding is acutely painful and induces strong behavioral and physiological responses in absence of pain mitigation [29]. There is a growing body of evidence indicating that the intra-operative pain can be controlled by using general anesthesia, local anesthesia or a combination of both [30,31]. However, effects of these drugs are limited to approximately 2 hours and, therefore, fail to mitigate the post-operative pain [28,31,32]. Evidence of post-operative pain begins when the effects of the drugs wane as shown by behavioral and physiological changes [31]. There is also some evidence indicating that the post-operative pain is associated with persistent negative affective states. Calves experiencing hot-iron disbudding show conditioned place aversion (i.e., aversion of the room they have been dehorned in; [33]) and express depressive-like behaviors such as negative judgment bias [34,35] and anhedonia (a reduced ability to experience pleasure; [36]).

Post-operative pain associated with disbudding can be mitigated using analgesics such as Non-Steroidal Anti-Inflammatory Drugs (NSAID) [31]. A recent study showed that providing an NSAID renders the procedure less aversive [37], suggesting that a multi-modal pain mitigation strategy may dampen the negative affective experience associated with the procedure, at least for the first day after the procedure. However, these drugs only mitigate the pain for hours to days (depending on the NSAID); whereas, the pain associated with disbudding has been reported to last for weeks [38,39]. These lines of evidence show that pain mitigation helps but does not completely suppress the pain associated with these procedures.

According to the EU organic regulations, some forms of pain mitigation (anesthesia or analgesia or a combination of the two) must be provided (see Table S1, Supplementary Materials). It is unfortunate that the EU organic regulations fail to require multi-modal pain control; however, the mandatory requirement to use some form of pain control is encouraging. A complete elimination of the pain induced by disbudding would only be achieved by banning this procedure, by developing new pharmacological ways of controlling the long-lasting pain or by breeding for polled cattle (reviewed by [40,41]). Achieving hornlessness through genetic modifications has also been documented [42,43]. However, genetically modified organisms are forbidden in EU organic systems and largely regulated in conventional systems and the absence of knowledge regarding potential consequences for animals render this option unlikely to be explored further within the EU.

A complete ban on dehorning/disbudding would require changes in the current management of cattle, particularly under confinement housing systems. Horned cattle maintain higher inter-individual distances and have lower physical interactions compared to hornless cattle [44], suggesting that higher space allowance and absence of competition for feed would prevent horn-related issues. However, comparison of different types of rearing conditions is fraught with challenges [45]; a fact that must be considered given that horned and hornless cattle are often raised in very different environments, with horned cattle often housed under more extensive systems than indoor housed cattle that are often hornless. Management of horned cattle will require adaptions that take into consideration cattle species-specific needs to reduce occurrences of horn-related injuries. This should be achievable given the low levels of injuries observed when managing horned cattle in some farms [46].

## 3. Calf Care

### 3.1. Social Housing

For the purpose of this review, we will consider social isolation as the absence of full physical contact with peers. Within the dairy industry, issues pertaining to social isolation are focused almost exclusively on dairy calves. Within the EU, on conventional farms, dairy calves can be housed individually until they are 8 weeks of age (see Table S2, Supplementary Materials). In contrast, the period of individual housing in the EU organic regulations is limited to the first week of life.

Given that cattle are herd animals; social contact is a fundamental behavioral need. Individually housed calves show social [47], emotional [48,49] and cognitive deficits [50,51] compared to calves housed in social groups. In addition, calves reared in social groups show less food neophobia [52,53] and have been found to eat twice as much solid feed and to show less distress during weaning compared to single housed calves [54,55]. Limiting social isolation to the first week of life is clearly an improvement given that calves separated from the dam at birth start interacting with other calves as soon as the second day of life [56]. In addition, young calves are motivated for full social contact [57] and prefer familiar individuals [58,59], suggesting the early establishment of social bonds as important and long lasting [60].

One point overlooked by the current EU organic regulations is group composition. Group size and whether group composition is dynamic matter; small stable groups (<8–10 calves) show benefits in terms of health and reduced competition (reviewed [61]). Competition in group housed calves for access to teats is reduced when more teats are provided [62], or by reducing the number of calves accessing the same automatic milk feeder [63]. Given the negative effects of larger group sizes on health [61], we suggest that group size should be kept small (<10 calves), that competition for teats should be reduced and that stable groups should be favored to reduce social stress and disease [64].

Advocates of individual housing argue improved calf health through reduced opportunity for disease transmission between individuals [65]. However, the overall evidence is mixed as some studies report no differences in health status when comparing individual versus small groups of calves [66,67] or improved health in group housed calves [68,69]. Some argue that group housing increases the risk factor for cross-sucking, an abnormal and repetitive behavior where a calf suckles the body of another, often the udder [70]. This behaviour is absent when calves are reared with their dam or with a nurse cow [71,72] and rarely observed in calves fed higher volumes of milk through a nipple [70]. We turn to methods of care that mitigate cross sucking in the next section.

### 3.2. Feeding

Calf feeding practices outlined in the organic regulations focus on the feeding of whole milk for the first 3 months of life (see Table S3, Supplementary Materials). Unfortunately, the regulations are silent on the type of milk feeding system (teat versus bucket feeding), the total daily volume provided and how best to transition the calves from milk to solid feed (wean).

Historically, dairy calf feeding management practices have promoted feeding ~10% body weight equivalent of milk, which is considerably less compared to when the calf is able to suckle from the dam. In the US, it is still common for calves to be bucket fed between 4 and 6 L of milk per day (USDA, 2016). In contrast, when Holstein calves are provided *ad libitum* access to milk, they drink on average 11 L to 16 L per day in the first 6 weeks [73]. Feeding higher volumes of milk has been shown to provide clear benefits in terms of calf health, weight gain [74] and milk production later on in life [75]. In the EU, the recent available literature on calf feeding studies often still includes a ‘control’ treatment of 4–6 L, likely reflecting standard industry practices (i.e., Denmark: [76]; Spain: [77]). Feeding low volumes of milk reduces average daily gain, increases behavioral signs of hunger [78,79], and increases cross-sucking [61,80]. In addition, nipple feeding systems promote natural sucking behavior [81,82,83], increase levels of insulin and cholecystokinin [84], and reduce cross-sucking (see reviews [61,70]). We strongly encourage that the next revision of the organic regulations be explicit in how much milk calves should be provided on a daily basis, encouraging *ad libitum* milk intakes fed using a nipple.

The EU organic regulations explicitly state that restricted feeding is forbidden unless justified for veterinary reasons. Unfortunately, the regulation is unclear as to what “restricted feeding” refers to. We strongly urge clarity on this point. For instance, dairy cattle are frequently fed restricted amounts of feed during the pre-reproductive (fed a low energy diet; [85,86]) and dry-off periods (fed a low-energy diet to reduce milk production; see review by [87]). Unfortunately, the language used within the EU organic regulations is vague as to the status of these practices; we encourage future regulations to clearly prohibit all feeding practices that potentially lead to hunger.

Dairy calves are weaned from milk at much earlier ages than when allowed to suckle from the dam (see review [88]). Early and/or abrupt weaning from milk has been shown to induce weight loss, counterbalancing the increased average daily gains observed when feeding higher volumes of milk [89]. Abrupt removal of milk has also been shown to trigger emotional distress and increase cross-sucking among calves [90]. Unfortunately, there is little guidance within the literature as to how long calves should be provided milk before weaning. Given that in nature calves would spend 7–9 months suckling their dam [88], the stipulation in the EU organic regulations that calves be weaned no earlier than 3 months of age is an improvement compared to the conventional regulations that are silent on this aspect of calf rearing. Regardless of age, weaning is a stressful event for calves (see review [91]) and every effort should be made to avoid abrupt weaning; gradual weaning that promotes solid feed intake reduces weaning distress [90,92]. Unfortunately, the organic regulations have failed to address this issue and so we encourage future regulations to promote gradual weaning to avoid unnecessary distress, weight loss and the expression of abnormal behaviors.

The EU organic regulations also state that roughage must be included in the calves’ diet. This is consistent with the onset of grazing behaviors (starting around 3 weeks of age) observed when calves are reared on pasture [93]. Recent research also indicates that calves provided access to forage when fed high volumes of milk and calf starter show improved rumen development and higher rumen pH compared to when they are not provided forage [94].

The Council directive for calves [27] states that water must be provided no later than 14 days of age, when they are sick and in hot weather conditions (see Table S4, Supplementary Materials). In contrast, many industry led groups (e.g., the Canadian Code of Practice for the Care and Handling of Dairy Cattle, [95]) requires that all cattle be provided access to clean and palatable water; this practice is supported by work indicating that provision of water beginning immediately after birth benefits calves’ development [96]. Moreover, we speculate that calves kept in hot climates would also have a greater need for water during the first 14 d of life where water provision is not mandatory. Overall, despite some modest improvements, the organic regulations fail to provide clear guidance on the provision of milk, solid feed and water.

## 4. Indoor Housing

### 4.1. Lying or Resting Area

Providing “a comfortable, clean and dry laying or rest area” is viewed as a necessity in most dairy cattle care guidelines [95]. At best this should be the minimum requirement given that dairy cattle can spend on average 9–13 h a day resting; a behavior that they are highly motivated to engage in [97,98,99]. Unfortunately, the organic regulations provide little additional guidance on what is required for the resting area in cattle (see Table S6, Supplementary Materials).

In contrast to the regulations for conventional farming (see Table S7, Supplementary Materials), EU organic regulations explicitly forbid tethering. However, small organic holdings (maximum 50 animals) can seek an exemption if cows have access to pasture during the grazing period and have bi-weekly access to open areas for exercise during non-grazing periods. Clearly, access to an exercise area following any period of tethering is likely valuable for tied cows [100]. However, to our knowledge, there is no available evidence suggesting that biweekly access is sufficient to ensure cow welfare.

Most of the research to date on tie-stall systems has focused on physical health indicators. For example, although some showed cows housed in tie-stalls have an increased prevalence of heel erosion and hock injuries compared with cows housed in a loose system [101,102]; others have shown reduced prevalence of claw lesions [103]. Restriction of movement is usually assumed to trigger negative affective states. For instance, pigs subjected to repetitive periods of short restraint expressed depressive-like states [104]. Unfortunately, there is little work on the effect of tethering on affective states in cattle. Considering that cows “have a behavioral need for locomotion” [105] and the public’s concern with long durations of tethering [106], we predict that this tie-stall system will receive growing criticism as public awareness grows. In addition, some members of a Panel on Animal Health and Animal Welfare stated that “there is sufficient evidence of poor welfare in dairy cattle held in tie-stalls” and “recommended that dairy cattle should not be routinely kept in tie-stalls as a housing system” (EFSA, 2009). The practice is indeed unlikely to satisfy “high standards of animal welfare”, as stated in the organic regulations. Thus, although the ban of tethering dairy cattle by the organic regulations is an improvement, the exception regarding small holdings is problematic.

With the exception of tethering discussed above, the EU organic regulations have stayed silent on other types of housing (freestalls or open/bedded packs). Instead they provide basic guidelines on housing features that are common amongst systems. For instance, straw bedding but also “[other] suitable natural material” such as sawdust and sand, as long as it is “dry”, are allowed. Wet bedding dramatically decreases lying time for both adult cows [107,108] and calves [109]. Unfortunately, there is no guidance as to what “dry” means nor in the quantity of bedding; the litter shall be “ample.” This is concerning given that cows housed in freestalls prefer stalls with more bedding [110,111]. The regulations also provide no information on the benefits associated with deep-bedded freestalls systems compared to mattress or rubber mat stalls. According to the available research, sand-bedded freestalls should be viewed as the gold standard (reviewed by [112]); deep-bedded systems are associated with reduced lameness, hock injuries [113,114,115] and mortality [116].

The literature on cow behavior in freestalls is dense and many studies have explored different aspects of the freestall environment on behavior and health, such as how cows react to certain aspects of stall design. Freestalls were originally designed to provide cows with an area to lie down but to prevent them from standing fully inside the stall, using a neck rail and/or brisket board. However, cows prefer stalls without brisket boards [117] and spent less time perching (i.e., standing with the two front hooves in the stall) when neck rails were less restrictive [118]. Thus, by preventing cows from standing fully in the stall, freestalls with neck rails favor standing with the hind legs on concrete, a surface associated with increased risk of lameness, hoof problems, leg fractures and claw damage [119]. However, neck rails improve hygiene and udder cleanliness, thus, reducing the risk of subclinical mastitis [120]. In conclusion, when cleanliness of the stalls and consequently, of the cows is prioritized, standing behavior is altered. Some authors “recommend that producers avoid restrictive neck rails so that cows can use the stall as a dry and comfortable standing area, although this will likely require more frequent stall cleaning” [121].

The status of freestalls housing systems with the EU organic regulations is difficult to evaluate. According to the organic regulations, cows should be provided “with sufficient space to stand naturally, lie down easily, turn around, groom themselves, assume all natural postures and make all natural movements”; a provision that cannot be guaranteed when cows are housed in freestalls. Housing cows in freestalls forces cows to lie down in a specific way, restricts their freedom of movement and hinders natural behavior [122]. Thus, open bedded packs may be a more appropriate housing system as they allow cows “to assume all the natural lying positions” [123].

Some research has explored the welfare benefits associated with one type of dairy housing compared to another (i.e., freestalls versus open packs). Others, however, have cautioned against such comparisons given the different challenges observed with different types of housing systems [15]. For instance, open packs (i.e., straw yards and compost-bedded pack) are often associated with reduced hygiene, particularly at low space allowance [124]. In contrast, open packs have been shown to present advantages such as increased lying behavior and lying synchrony ([125,126]; see review on compost-bedded packs [127]). When given the choice between freestalls and an open pack, cows showed a “small” preference for lying down in the open pack but a “strong” preference to stand with all four hooves in the open pack [126]. Cows also showed some play behavior—a marker of positive emotion [128]—when initially moved from free stalls to an open pack [125]. Finally, open packs may favor socio-positive behaviors in cows [129]. Thus, although open areas are more challenging to keep clean, they provide some welfare benefits.

### 4.2. Stocking Density

Although EU organic regulations require a minimum space of 6 m^2^ per cow, they do not differentiate between different resources such as the lying area or the feeding area (see Table S9, Supplementary Materials). Space requirements for cows housed indoors has received little attention in the scientific literature. The 6 m^2^/cow recommended is low when compared to other jurisdictions such as the 11 m^2^/cow when housed on a bedded pack in the Canadian organic standards [130].

Numerous studies undertaken in freestall facilities under zero grazing conditions report negative effects of overstocking dairy cows. For instance, overstocking in freestalls induces decreased lying time [107,131,132,133] and increases hock injuries [113]. In contrast, one study comparing 9 m^2^ and 4.5 m^2^ per cow on an open pack system, reported no differences in lying time [124]. Higher stocking density may be less detrimental in open packs compared to freestalls systems because all cows are able to “lie down at the same time staying closer to one another” [134].

Stocking density recommendations should be evaluated in terms of each key resource. For example, recent evidence indicates that cows will also actively compete for water, particularly in hot weather [135] and for access to fresh feed [136,137]. Installation of a headlock feed barrier or feed stalls can reduce the rate of competitive displacements, compared to more open types of feed barriers [136,138,139]. Regardless, all animals should be provided sufficient feed space to allow all group-members to feed at the same time, particularly during vulnerable periods such as before and after calving. Unfortunately, the current EU regulations on organic farming do not provide any guidance regarding feeding space.

Other factors can also contribute to the negative effects of overstocking such as group size, group instability (i.e., frequent introduction of new animals into the group) or social dominance. For instance, the negative effects of regrouping are enhanced when done at higher densities [140] and such effects can be detrimental at calving [141]. For instance, multiparous cows were associated with an increased risk of metritis (i.e., an infection of the uterus following parturition; [142]) after calving when housed in an unpredictable and competitive environment before calving [143]. In addition, when overstocked, low-ranking cows spend less time lying down and more time standing in the alleys and are consequently more susceptible to lameness compared to middle- and high-ranking cows [144]. Cows with high milk yield and a low space allowance were less clean and showed higher incidence of mastitis than cows with a higher space allowance [124].

We encourage the EU law-makers to address the challenges associated with overstocking by adopting more precise and bold regulations in order to avoid excessive competition for access to key resources.

## 5. Outdoor Access

According to the EU organic regulations, dairy cows must have access to pasture for grazing. Exceptions exist regarding weather conditions, state of the ground and obligations related to the protection of human and animal health. However, there is a lack of precision regarding the time (i.e., How many days per year? How much time per day?) that cows must spend on pasture. The regulations only state that they must have access to pasture “whenever conditions allow”. This lack of precision can jeopardize the intentions of the regulations.

In addition, although tie-stalls systems must provide pasture during the grazing period they must also “provide open air areas during the winter months” twice per week. Yet, the organic regulations are vague as to the type of outdoor access or open-air area (i.e., hard or soft flooring; type of materials) and the duration cows should spend outside. EU law-makers also did not extend outdoor access during the non-grazing months to all cows regardless of housing type. Providing an outdoor open bedded pack with soft flooring may be an effective alternative to pasture when the weather prevents access to pasture (for a review, see [145]).

Recent research indicates that pasture access is valued by cattle. The EU organic regulations clearly specify that cattle must have access to pasture; a position that is in line with their preference for a large pasture compared to a small sand pack [146], suggesting that space and/or grazing may be an important component of cows’ motivation to be outside. For pastured cows, the available evidence suggests that they spend ~68% of their time grazing [4]. When given the choice, dairy cattle show a strong preference for pasture compared to indoor housing (reviewed by [147,148]), especially during the night [149]. However, preference for pasture is affected by weather conditions. When given the choice between pasture and freestalls, cows spent less time on pasture during rainy days [149,150,151], winter [152,153] and when the temperature-humidity index (THI) was high during the day (i.e., beyond the thermal comfort zone for cows; [149]). Although preference studies provide insights on what resources animals choose in different situations, they fail to provide information on how important the resource is to them [154]. von Keyserlingk et al. [5] found that cows value access to pasture as much as fresh feed (viewed as a gold standard for assessing motivational states), particularly during the late afternoon and evening hours, illustrating the importance of pasture access for dairy cows.

Although cows prefer and are motivated to access pasture, some studies report that cows housed in pasture have lower body condition scores, body weight and higher endoparasites infection [155]. In contrast, there is evidence that pasture provides benefits such as reduced mortality, lameness, hoof pathologies, hock lesions, mastitis and metritis (reviewed by [147]). These pathologies challenge the quality of cattle’s life and longevity and induce pain and suffering. For instance, lameness [156], poor claw conditions [157], mastitis [158] and metritis [159] have all been shown to induce pain in dairy cows. Lameness [160], mastitis [155] and metritis [161] have also been associated with earlier culling.

Fortunately, EU law-makers have addressed the issues associated with adverse weather conditions. According to the latest organic regulations, “shelters or shady areas” must be provided (see Table S11, Supplementary Materials), which is consistent with data showing that cows (that usually go outside during the Summer) are more affected by heat than by cold conditions (reviewed by [162]). When shade is provided, it is used by cows during hot days and can mitigate heat stress ([163]; for a review on the effects of heat stress, see [164]). The importance of shade during hot days is clear as cows will choose to stand in shade rather than lying down in the sun even after 12 h of lying deprivation [165]. In addition, according to the organic regulations “Organic livestock rearing in a pen on very wet or marshy soil shall not be allowed”, which is supported by recent work showing cows’ aversion for muddy conditions [166].

Although somewhat unclear (see Tables S10–S12, Supplementary Materials), the organic regulations state that calves and heifers should be provided with outdoor access whenever conditions allow. We presume that outdoor access is mandatory when calves are being regrouped with one or multiple other calves (>1 week of age; see Section 3.1) but greater clarity is needed to avoid confusion given that it is unclear what is meant by an outdoor area and at what age this requirement should be enforced.

There is a dearth of research on outdoor access for calves. In a recent study, Wormsbecher et al. [167] housed calves (as single or as pairs) in individual hutches with an outdoor access. Although this study should be viewed with caution given the confound regarding space per calf, the authors reported that all calves spent time outside the hutches during both Winter (47.5% for paired calves; 48.7% for single calves) and Summer (58.7% for paired calves; 75.7% for single calves), a result indicating that they may value outdoor access. More work is needed to understand whether calves are motivated to access the outdoors, and the effects of different types of outdoor access on their development.

## 6. Health

Ensuring that animals are in a good health or “function well” [168] is an essential component of animal welfare and has been argued by some to be undermined in organic livestock production [169]. Thus, recommendations that collectively decrease the occurrence of diseases, promote early diagnosis and efficient treatments are a key component of welfare. The focus of EU organic farming to prohibit use of synthetic chemicals for veterinary use, with the exception of vaccines and anti-parasites, requires that the industry move towards prevention of maladies [170,171]. However, unlike the US organic regulations which prohibits antibiotics, the EU organic regulations allow their use but under much more restrictive guidance compared to conventional farm (see Table S13, Supplementary Materials). For instance, antibiotics are to be used only when alternative ‘non-antibiotic’ treatments have failed (see Table S13, Supplementary Materials) and must be approved by a veterinarian; and no animal must receive more than three treatments per year. Since implementation of these restrictions, anti-microbial use has declined dramatically on organic farms in the EU [172].

We see two potential consequences of such regulations on the welfare of the animals. First, farmers are encouraged to use alternative methods/treatments and adopt pro-active preventive solutions to reduce disease prevalence. This may lead to either no changes or to moderate improvements in overall health compared to conventional systems. However, by promoting a culture of ‘last resort antibiotic use’ and the use of alternative treatments of sometimes unknown efficiency, organic regulations may in some cases prolong treatment, thereby, causing prolonged suffering [8,169,173].

Some studies indicate that organic dairy farmers have changed their management routines to facilitate disease prevention. These changes include providing outdoor access, improved bedding hygiene, straw bedding and increased emphasis on udder health during milking [171]. In addition, some organic dairy farmers pay closer attention to early signs of diseases [174], leading to earlier intervention and increased care [175]. Some farmers also report keeping antibiotics on hand for cases where symptoms get worse or when animals fail to show signs of recovery in a timely manner [176].

Assessing the efficacy of different systems on animal health is challenging [45]. Some studies have recorded the number of treatments given to animals as a proxy health measure, with some evidence suggesting an improved herd health in organic productions compared to conventional farms [177]. However, this measure should be viewed with caution given that simply reporting fewer treatments does not necessarily equate to lower incidence of disease given the potential confound of different thresholds for treating sick animals due to longer withdrawal periods [172,178]. In addition, although the use of conventional treatments is generally recorded by veterinarians, alternative treatments may not be systematically reported; this may artificially decrease the number of treatments recorded in organic farms [175].

When comparing health status of cows in conventional and organic systems, most studies have relied on observational data with few controlled experimental studies (see review [179]). Results vary greatly across studies, making it difficult to draw clear conclusions. In studies comparing conventional to organic, the majority of studies have focused on prevalence of mastitis [169] with organic dairy cows having higher rates of subclinical mastitis [180,181]. However, this result is clearly dependent of whether or not organic farmers chose to keep using antibiotics at all. Organic farms that chose to abolish the use of antibiotics had higher somatic cell counts (a proxy measure of poor udder health and of subclinical mastitis) compared to conventional farms, but no differences were found between Spanish organic farms that use antibiotics (when needed) and conventional farms [182]. These results confirm previous work showing either no differences in mastitis prevalence or treatments between both systems [171,183] or reduced prevalence of mastitis in organic herds [175,184]. Low mastitis rates during lactation may be due to less intensive milk production in the organic systems, reducing the udder vulnerability to pathogens [175,185]. A study in the UK found higher risk of mastitis during the dry period (period where animals do not produce milk) probably due to the absence of prophylactic antibiotics (for contrasted results, see [170]); the reader should be aware that, with few exceptions (i.e., The Netherlands), it is common practice to preventively treat conventionally raised cows with antibiotics just before the dry period [186]. The reduced use of antibiotics does not seem to negatively impact dairy cows [187]. However, although some studies found culling rates to be lower in organic farms, udder health remains one of the top cited reasons for culling on these farms (along with fertility and foot problems similar to what is seen in conventional systems; [176,188]). Future work should focus on other health parameters, as most of the studies have neglected other infectious diseases. Some studies have reported a reduced occurrence of pneumonia in grazing herds, regardless of whether they were organic or conventional compared to indoor housed herds [184]. No differences in farm system type were found in the number of treatments for endometritis [176].

A more cautious approach to the use of antibiotics could potentially have long-term beneficial effects on drug efficiency, which can ultimately have positive effects on the welfare of dairy cows. For instance, some studies show a decrease in antimicrobial resistance in farms that have transitioned from conventional to organic ([179,189] but see contrasting results [190]). However, farms that do not use antibiotics are not exempt from udder health issues [182], highlighting the importance of keeping antibiotics in the list of available treatments in organic dairy farms; a view that is shared by some organic dairy farmers [171].

There are a few reports indicating lower [177,183,184] or no differences in metabolic diseases [175] such as ketosis (the abnormal increase in blood ketones levels due to liver issues at the beginning of lactation). This may be explained by lower milk production, which is a risk factor for this pathology [184]. Milk fever (important decline in blood calcium concentration following calving) has been reported to be lower in organic dairy farms [176].

The available data illustrates the high variation that exist regarding animal health status in different farms (organic or non-organic). This shows the difficulty in relying on input-based measures that aim at improving animals’ environment to efficiently tackle health issues in dairy farms [191]. Organic dairy farmers generally agree that regulations should clearly state minimum requirements regarding animal health [191]. To improve health in organic herds, we recommend that in addition to the existing facility (i.e., input) based measures (i.e., flooring type), animal (i.e., outcome) based measures (e.g., severe lameness prevalence) be incorporated into the legislation. For instance, in the case of severe lameness, a specific target threshold (e.g., no more than 5% of the lactating herd can be scored as severely lame at any given time) should be required. This required minimum standard for animal-based measures should result in improved welfare but also provide the opportunity for the producers to identify tailored solutions that work on their farm, enabling them to achieve this objective.

## 7. Post Farm Gate

Although this review focuses primarily on on-farm aspects, we briefly highlight the little emphasis the EU organic regulations place on the welfare of the animals once they leave the farm (i.e., transportation and slaughter). With the exception of two arguably vague statements (i.e., minimizing the duration of transport and avoiding or keeping to a minimum any suffering, pain and distress at the time of slaughter), there are only two specific provisions in the organic regulations that reference this part of the production process (i.e., ban of allopathic tranquilizers prior to or during transport for animals coming from organic farms and any type of electrical stimulation during loading and unloading). Apart from these minor provisions, the Council regulation n°1/2005 “on the protection of animals during transport and related operations” [192] and the Council regulation n°1099/2009“on the protection of animals at the time of killing” [193] governs what happens to farm animals, regardless of whether they come from an organic or a conventional farm.

These two regulations incorporate some rules, albeit limited, aimed at protecting the animals during transport, lairage and during slaughter (e.g., the staff has to be trained; unfit animals cannot be transported; required standard operating procedures; priority to lactating dairy animals at the slaughterhouse; for more information, see Tables S14–S18, Supplementary Materials). Regarding transportation, the absence of specified maximum journey durations is worrisome. Although journey time shall not exceed 8 hours, as currently written the text can be interpreted to allow the journey to be extended if certain requirements are met, specifically if some breaks are provided during the journey (see Table S17, Supplementary Materials). Although more research is needed in this area, it is known that long transport, especially during adverse climatic conditions, can seriously challenge the welfare of cattle [194,195]. Therefore, consistent with its aim to reduce the duration of transport, we regret that the current organic regulations did not include a maximum journey time.

The current transportation regulations and the lack of enforcement have raised concerns among the public [196]. These concerns are shared by some, including the European Parliament who have urged the Member States—twice over the last two years—to better enforce the existing EU rules on transportation. The European Parliament has also called for reducing the duration of transport and, ultimately, the trade of live animals [197,198]. Some alternatives to live animals’ transport have been explored, such as on farm or local slaughter, which may mitigate some of the issues associated with transportation [199,200,201]. We strongly encourage future research to explore these different alternatives and the organic regulations should, in the future, encourage the use of such alternatives.

Similar observations can be made about slaughter rules within the EU, as the EU law-makers fail to provide any additional provisions in the organic regulations to what was cited in the 2009 slaughter regulations. However, despite the absence or the vagueness of the organic regulations on the matter, the European Court of Justice (CJEU) recently ruled on the compatibility between organic farming and ritual slaughter without stunning, a practice that is permitted in the EU under the right to freedom of religion [202,203,204].

The CJEU was asked to consider whether slaughter without stunning was in line with the objective of ensuring high animal welfare standards stated in the organic regulations. For the European Court, slaughter without stunning is “not tantamount, in terms of ensuring a high level of animal welfare at the time of killing, to slaughter with pre-stunning which is, in principle, required”. Stunning is indeed supposed “to induce a lack of consciousness and sensibility before, or at the same time as, the animals are killed” [193] and—when correctly performed –, prevent pain and distress during slaughter [205,206,207,208]. Thus, according to the Court, the practice of slaughtering without stunning animals fails to observe the highest animal welfare standards and thus deemed to be incompatible with the organic regulations (see Table S18, Supplementary Materials). Readers should be aware that the court judgement does not prevent consumers from being able to purchase products coming from animals killed without being pre-stunned. However, the court has ruled that these products can no longer be marketed as organic.

These are only a few examples of welfare related-issues associated with transportation and slaughter (for a review on the stress associated with pre-slaughter conditions, see [209]). However, it must be noted that with the exception of the mandatory stunning before slaughter and the two aspects related to transportation mentioned above, organic cattle are only afforded the same protection as conventional farm animals once they leave the farm.

## 8. Conclusions

The EU organic regulations have provided additional rules that promote animal welfare on a number of issues compared to what is required under the EU regulations for conventional farming (e.g., pasture access; social housing for calves; no tethering; no physical mutilations). Given that organic farms must be visited on a regular basis, these changes have the potential of improving the lives of dairy cattle housed on organic farms. However, some limitations remain, including a number of instances that allow exceptions (e.g., disbudding; tethering), promoted in large part to the continued use vague language (e.g., calf care, indoor housing) or remaining silent on key issues known to impact dairy cattle welfare (i.e., both at the farm and on post farm gate). Future organic regulations should continue to focus on improving the welfare standards; this approach will hopefully also place pressure resulting in improvements in the standards for conventional farming. We also encourage future work to investigate the effectiveness of welfare regulations to improve farm animal welfare; an area that remains understudied.

## Supplementary Materials

The following tables displaying EU regulations related to the welfare of dairy cattle are available online at https://www.mdpi.com/2076-2615/10/10/1786/s1, Table S1: EU regulations related to physical mutilations (tail-docking, dehorning, disbudding), Table S2: EU regulations related to isolation, Table S3: EU regulations related to feeding, Table S4: EU regulations related to water access, Table S5: EU regulations related to housing – materials, lighting, ventilation and cleaning, Table S6: U regulations related to housing – lying or resting area, Table S7: EU regulations related to freedom of movement – tethering, Table S8: EU regulations related to housing – flooring, Table S9: EU regulations related to housing – stocking density, Table S10: EU regulations related to outdoor access, Table S11: EU regulations related to outdoor – characteristics, Table S12: EU regulations related to open air area (=excluding pasture) – density, Table S13: Main features of the EU regulations related to health, Table S14: EU regulations related to transportation – fitness for transport, Table S15: EU regulations related to transportation – means of transport, Table S16: EU regulations related to transportation – loading, unloading and handling, Table S17: EU regulations related to transportation – watering/feeding intervals, journey times, resting periods, Table S18: Main features of the EU regulations related to slaughtering, Table S19: EU regulations related to breeding – reproduction, Table S20: EU regulations related to staffing, Table S21: EU regulations related to inspection, Table S22: EU regulations related to handling.
